# Supplementary material for: Impact of Subsyndromal Delirium Occurrence and Its Trajectory during ICU Stay
Source: J Clin Med. 2022 Nov 17;11(22):6797. doi: 10.3390/jcm11226797 (PMC9692318; doi:10.3390/jcm11226797)
Supplement: Supplementary file 1 [file jcm-11-06797-s001.zip › Supplementary material SSD -JCM.pdf]

Supplementary material S1

Characteristics	Univariate logistic models		Multivariate logistic model		
	OR	p-value	OR_{aj}*	CI (95%)	p-value
**Mental status**		< 0.001			0.79
Normal	1		1		
SSD who susteined or improved	1.02	0.937	1.32	0.83 – 9.27	0.426
SSD who progress to delriium/coma	2.19	0.034	1.38	0.56 – 20.73	0.508
Delirium /Coma	4.61	< 0.001	1.48	0.96 – 10.86	0.988
**Mechanical Ventilation**					
non-ventilated	1		1		
mechanically ventilated	14.92	< 0.001	7.42	4.64 - 12.38	< 0.001
**Diagbnostic of admission**					
Surgical	1		1		
Medical	6.61	< 0.001	3.73	2.48 - 5.71	< 0.001
**Gender**					
Female	1		1		
Male	1.09	0.582	0.98	0.96 – 3.86	0.908
**Benzodiazepin use**					
No	1		1		
Yes	2.73	< 0.001	1.08	0.96 – 3.86	0.732
**Age**					
<50 yo	1		1		
50 - 69 yo	1.71	0.008	1.49	0.86 - 2.56	0.151
>70 yo	1.56	0.04	1.78	1.00 - 3.25	0.057
**Apache II**					
<24 points	1		1		
>24 points	2.75	< 0.001	1.17	1.07 - 9.25	0.465

*OR_{aj} = *Odds Ratio* adjusted

Supplementary material S2

CAM-ICU feature	Total		Cognitive trajectory				P-valor (teste χ^2)
			Improved or stable		Worsened		
	n	%	n	%	n	%	
**Flutuation**							
No	69	100,0	47	68,1	22	31,9	0,729
Yes	34	100,0	22	64,7	12	35,3	
**Attention deficit**							
No	68	100,0	45	66,2	23	33,8	0,807
Yes	35	100,0	24	68,6	11	31,4	
**Disorganized thinking**							
No	78	100,0	55	70,5	23	29,5	0,179
Yes	25	100,0	14	56,0	11	44,0	
**Level of consciousness**							
No	66	100,0	46	69,7	20	30,3	0,435
Yes	37	100,0	23	62,2	14	37,8	
